# A Mutation in *CsYL2.1* Encoding a Plastid Isoform of Triose Phosphate Isomerase Leads to *Yellow Leaf 2.1* (*yl2.1*) in Cucumber (*Cucumis Sativus* L.)

**DOI:** 10.3390/ijms22010322

**Published:** 2020-12-30

**Authors:** Liangrong Xiong, Hui Du, Keyan Zhang, Duo Lv, Huanle He, Junsong Pan, Run Cai, Gang Wang

**Affiliations:** School of Agriculture and Biology, Shanghai Jiao Tong University, 800 Dongchuan Road, Minhang District, Shanghai 200240, China; xlr1394981178@sjtu.edu.cn (L.X.); duhui1122@sjtu.edu.cn (H.D.); lnykzky2016@sjtu.edu.cn (K.Z.); lvcloudy@sjtu.edu.cn (D.L.); hlhe75@sjtu.edu.cn (H.H.); jspan71@sjtu.edu.cn (J.P.); cairun@sjtu.edu.cn (R.C.)

**Keywords:** cucumber, yellow leaf, chlorophyll metabolism, chloroplast development, ptTPI

## Abstract

The leaf is an important photosynthetic organ and plays an essential role in the growth and development of plants. Leaf color mutants are ideal materials for studying chlorophyll metabolism, chloroplast development, and photosynthesis. In this study, we identified an EMS-induced mutant, *yl2.1*, which exhibited yellow cotyledons and true leaves that did not turn green with leaf growth. The *yl2.1* locus was controlled by a recessive nuclear gene. The *CsYL2.1* was mapped to a 166.7-kb genomic region on chromosome 2, which contains 24 predicted genes. Only one non-synonymous single nucleotide polymorphism (SNP) was found between *yl2.1* and wt-WD1 that was located in Exon 7 of *Csa2G263900*, resulting in an amino acid substitution. *CsYL2.1* encodes a plastid isoform of triose phosphate isomerase (pdTPI), which catalyzes the reversible conversion of dihydroxyacetone phosphate (DHAP) to glyceraldehyde-3-phosphate (GAP) in chloroplasts. *CsYL2.1* was highly expressed in the cotyledons and leaves. The mesophyll cells of the *yl2.1* leaves contained reduced chlorophyll and abnormal chloroplasts. Correspondingly, the photosynthetic efficiency of the *yl2.1* leaves was impaired. Identification of *CsYL2.1* is helpful in elucidating the function of ptTPI in the chlorophyll metabolism and chloroplast development and understanding the molecular mechanism of this leaf color variant in cucumber.

## 1. Introduction

The Chloroplasts are plastids that contain chlorophyll and are the site of photosynthesis, the process by which light energy is converted to chemical energy, resulting in the production of oxygen and energy-rich organic compounds. In plants, chloroplasts occur in all green tissues, though they are concentrated particularly in the parenchyma cells of the leaf mesophyll. Chloroplasts are distinguished from other types of plastids by their green color, which results from the presence of two major types of pigments in higher plants and green algae, chlorophyll a (Chl_a_) and chlorophyll b (Chl_b_). The main function of Chl_a_ and Chl_b_ pigments is to absorb energy from light for photosynthesis [1]. Most photosynthesis products are stored in the seeds as storage reserves which will be used to support postgerminative seedling establishment until chloroplast and leaf development are complete.

Seed germination and seedling establishment are pivotal for the life cycle of seed plants. Storage reserve mobilization provides energy and carbon to support seedling development. In plants, after storage reserves are used, seedling establishment requires a transition from heterotrophic to autotrophic growth to sustain plant growth [2]. In *Arabidopsis thaliana*, the triose phosphate isomerase (TPI) was found to be involved in several metabolic pathways during this transition, including glycolysis, gluconeogenesis, and the Calvin cycle [3]. In *A. thaliana*, there are two genes annotated as TPI: one is cytosolic TPI (cytoTPI, *At3g55440*), localized to the cytoplasm; the other is the plastid isoform of TPI (pdTPI, *At2g21170*) localized to the plastid. Chen et al. discovered a knock-down mutant for pdTPI that revealed that pdTPI plays a crucial metabolic role during the heterotroph–autotroph transition phase, affecting both chloroplast development and seedling establishment in *A. thaliana* [4]. The loss of function of pdTPI prevented the conversion of dihydroxyacetone phosphate (DHAP) to glyceraldehyde-3-phosphate (GAP) in the chloroplast and resulted in DHAP accumulation and GAP decline in the chloroplast. The *pdtpi* mutant produced severely stunted and chlorotic seedlings that accumulated DHAP [3]. Chloroplast morphology and starch synthesis were also defective in the mutant [3]. These observations demonstrated that pdTPI plays an essential role in seedling establishment and chloroplast development in *A. thaliana*.

Cucumber (*Cucumis sativus* L.) is an economically important vegetable crop widely cultivated across the world. As part of photosynthesis, leaves play an important role in the growth and development of cucumber. Leaf color mutants are ideal materials for studying pigment metabolism, chloroplast development and differentiation, and photosynthesis. So far, many leaf color mutants have been reported but only five of them have been cloned in cucumber. Miao et al. identified *CsCNGCs* as the candidate gene for *virescent leaf* (*v-1*), which encodes a cyclic nucleotide-gated ion channel protein [5]. Gao et al. reported a *C528* mutant that exhibits golden leaves throughout the whole growth stage and identified *Mg-chelatase I subunit* (*ChlI*) as the candidate gene [6]. The cucumber *virescent-yellow leaf* (*vyl*) mutant has virescent, yellow-green young leaves, caused by a mutation in the *CsVYL* gene that encodes a DnaJ-like zinc finger protein [7]. The cucumber leaf color mutant *yellow-green leaf* (*ygl1*) was believed to be caused by a mutation in the *LOX* gene [8]. A mutation in *CsHD* encoding a histidine and an aspartic acid domain-containing protein leads to yellow young leaf (*yyl-1*) in cucumber [9]. The mutations underlying several of these leaf color defects have been cloned, but their molecular basis remains largely unknown. In this study, a new yellow leaf mutant induced by ethyl methane sulfonate (EMS) was identified from the wild-type inbred line wt-WD1 and was named *yellow leaf 2.1* (*yl2.1*). Here, we identified *YL2.1* by map-based cloning and showed that it encodes ptTPI in cucumber. This finding can help us to understand the underlying mechanisms of chlorophyll biosynthesis and chloroplast development.

## 2. Results

### 2.1. yl2.1, An EMS-Induced Mutant, Produces a Yellow Leaf Phenotype

**Figure 1:**
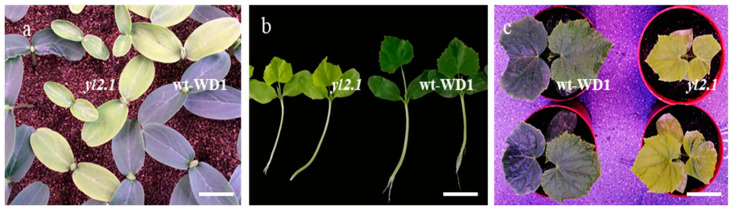
Phenotypical characterization of *yl2.1* and wt-WD1. Cotyledon color of the *yl2.1* and wt-WD1 (**a**); true leaf color of *yl2.1* and wt-WD1 at the first true leaf stage (**b**) and the two fully unfolded true leaves stage (**c**). Scale bar = 2 cm.

EMS is a stable and effective chemical mutagen. To facilitate functional genomic research in cucumber, a large number of cucumber mutant lines have been developed by the EMS-mediated mutagenesis procedure for cucumber in our laboratory. An EMS-induced mutant, *yl2.1*, was identified from the wild-type inbred line WD1 (wt-WD1) treated with EMS. The wt-WD1 is a North China-type inbred line of cucumber with normal green leaves. Compared with the wt-WD1, the *yl2.1* cotyledons were yellow in color after germination (Figure 1a). At the first true leaf stage of growing seedlings, the leaf color of the *yl2.1* was pale green (Figure 1b). When the seedlings grew to the second and third true leaf stage, the leaf color of the *yl2.1* gradually changed to dark yellow, but did not turn green as the leaves grew (Figure 1c), and the *yl2.1* seedlings exhibited reduced growth and lethality at the fourth leaf stage.

### 2.2. The Yellow Leaf Phenotype of the yl2.1 Was Controlled by a Recessive Nuclear Gene

Due to the seedling lethality of the mutant, the F_1_ can not be obtained by crossing 9930 (wild type North China fresh market-type cucumber, wt-9930) and the *yl2.1*. Then, the F_2_ population could not be obtained by the F_1_ self pollination. So, 10 plants with normal green leaves, including homozygotes (+/+) and heterozygotes (yl2.1/+), were pollinated to different plants of wt-9930 respectively to produce the F_1_. At the same time, the genotype of the 10 plants were identified by self pollination. The result showed that the leaves of three self-crossed progenies were green. Therefore, the three plants and their F_1_ crossed with wt-9930 were abandoned because the three plants were wild-type homozygous (+/+). The leaves of seven self-crossed progenies exhibited segregating phenotypes of green and yellow leaves, indicating the seven plants were heterozygous (*yl2.1*/+). All of F_1_ generations obtained by crossing these heterozygotes (*yl2.1*/+) and wt-9930 were retained to produce F_2_ population by self pollination. Two F_2_ populations with yellow and green leaves were selected for further analysis. To determine whether *yl2.1* was controlled by a single gene or by multiple genes, 1128 individuals of the F_2_ population derived from the cross between *yl2.1* and wt-9930 were used for genetic analysis. The 854 green wild-type individuals and 274 yellow mutants fitted a 3:1 segregation ratio (χ^2^ = 0.019 < χ^2^_0.05_ = 3.84). (Table 1). Thus, *yl2.1* was considered to be controlled by a single recessive nuclear gene.

### 2.3. Primary Mapping of the YL2.1

**Figure 2:**
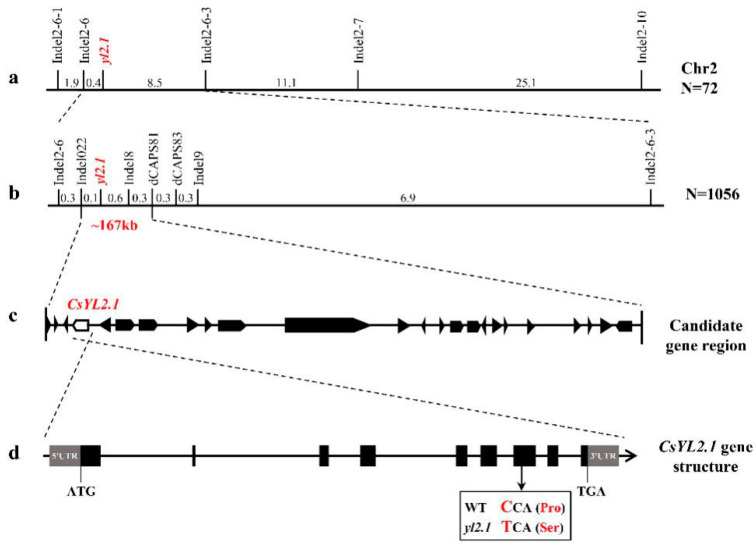
Map-based cloning of the *YL2.1* locus in cucumber. (**a**) Primary mapping with 72 F_2_ plants placed the *YL2.1* locus between InDel2–6 and InDel2–6–3 on cucumber chromosome 2. (**b**) Fine mapping with 1056 F_2_ plants narrowed down the *YL2.1* locus to a 167-kb region delimited by two markers Indel22 and SNP81. Numbers are genetic distances (cM) between adjacent markers. (**c**) Twenty-four genes were predicted in the 167 kb region; *CsYL2.1* (fourth) is the candidate gene. (**d**) Structure of the *CsYL2.1* candidate gene, which has nine exons. The SNP in the seventh exon makes the proline codon (CCA) mutate to a serine codon (TCA). The gray boxes, black boxes, and black lines indicate the untranscribed region, exons and introns, respectively.

Eighty polymorphic markers between the wt-9930 and the *yl2.1* were identified from 160 InDel markers distributed on seven chromosomes of cucumber. All 80 polymorphic markers between wt-9930 and *yl2.1* were used to analyze the W (wild-type phenotype) and M (mutant phenotype) DNA pools via the Bulked-Segregant Analysis (BSA) strategy. Among these markers, InDel2-7 and InDel2-10 were polymorphic between the W and M DNA pools. The two markers were located on chromosome 2 in cucumber. Genetic linkage analysis using 72 individuals of the F_2_ population showed that *YL2.1* was linked with InDel2-7 and InDel2-10 (Figure 2a). The result indicated that the *YL2.1* was located on chromosome 2. To further confirm the map location of *YL2.1*, 13 new InDel markers were developed on the basis of the resequenced genomic data. Among these markers, three markers were polymorphic between two parents and linked with *YL2.1*. The *YL2.1* was mapped between the markers InDel2-6 and InDel2-6-3 on chromosome 2 by genotyping 72 F_2_ individuals (Figure 2a).

### 2.4. Fine Mapping and Candidate Gene Analysis of the YL2.1

To fine-map the *YL2.1* locus, new markers were developed and tested for polymorphism between *yl2.1* and wt-9930. *YL2.1* was further mapped using the F_2_ population containing 1056 individuals. After characterizing the F_2_ population, *YL2.1* was mapped between the InDel markers InDel022 and InDel19, corresponding to a physical distance of 268.7 kb (Figure 2b). Within the 268.7-kb region, we tested 35 additional InDel markers, but no polymorphic marker was identified between *yl2.1* and the wt-9930. Two polymorphic dCAPs markers (dCAPS81 and dCAPS83) were developed on the basis of the SNP between the genomic sequences of *yl2.1* and wt-9930. Finally, *yl2.1* was mapped between the molecular markers InDel022 and dCAPS81, with an interval of 166.7 kb in physical distance (Figure 2b).

In the 166.7-kb genomic DNA region, 24 putative genes were predicted (Figure 2c). Detailed information on these genes are presented in Supplementary Table S1. According to the whole-genome resequencing data of *yl2.1*, only one highly reliable non-synonymous SNP was identified in these genes and their promoter regions (2000 bp before the start codon) between wt-WD1 and *yl2.1.* The SNP (‘C’ in wt-WD1 to ‘T’ in *yl2.1*) was found in the position 649 bp of the *Csa2G263900* coding sequence (Figure 2d and Supplementary Figure S1). Sequence analysis of the genomic DNA fragment of *Csa2G263900* further confirmed the SNP (‘C’ in wt-WD1 to ‘T’ in *yl2.1*) between wt-WD1 and *yl2.1*. The variation caused an amino acid at the 210th change from proline to serine (Figure 2d and Supplementary Figure S2). The results demonstrated that *Csa2G263900* was the most likely candidate gene for *YL2.1,* denoted *CsYL2.1.*

### 2.5. Analysis of the Putative Candidate Gene for CsYL2.1

The genomic DNA length of *CsYL2.1* (*Csa2G263900*) is 4564 bp and contains nine exons and eight introns (Figure 2d). The full-length cDNA of *CsYL2.1* (*Csa2G263900*) was 1177 bp in both wt-9930 and *yl2.1* and contained a 921 bp coding sequence and 256 noncoding regions (Figure 2d). *CsYL2.1* encods a 33-kDa protein of 306 amino acid residues. A protein BLAST (BLASTP) search for the *CsYL2.1* protein indicated that *Csa2G263900* encodes a predicted plastid isoform of triose phosphate isomerase (pdTPI). Besides, we use SuSPect (http://www.sbg.bio.ic.ac.uk/) analysis showing that the 210th Proline might be structural, and likely functionally, important (Supplementary Figure S3). In the Secondary structure analysis of protein, we found that when amino acid changed from proline to serine, causing “Membrane Interaction” and “Transmembrane Helix” turn into “Helix”, which means that the mutation of the Proline could destabilize the nearby alpha helix on one external side of the protein (Supplementary Figures S4 and S5).

To better understand the genetic and functional relationships of *CsYL2.1* between cucumber and other species, 14 putative orthologous proteins in other plant species were selected for further analysis (Supplementary Table S2). The alignment analysis revealed that amino acid sequence identity between CsYL2.1 and other orthologs varied from 97.7% (melon ortholog) to 71.6% (maize ortholog). The alignment showed that the amino acid sequences of CsYL2.1 and its orthologs are highly conserved in plants. The alignment also showed that only the YL2.1 protein of the mutant has serine, but the proteins of WT and its orthologs have proline at the SNP position (Supplementary Figure S6).

**Figure 3:**
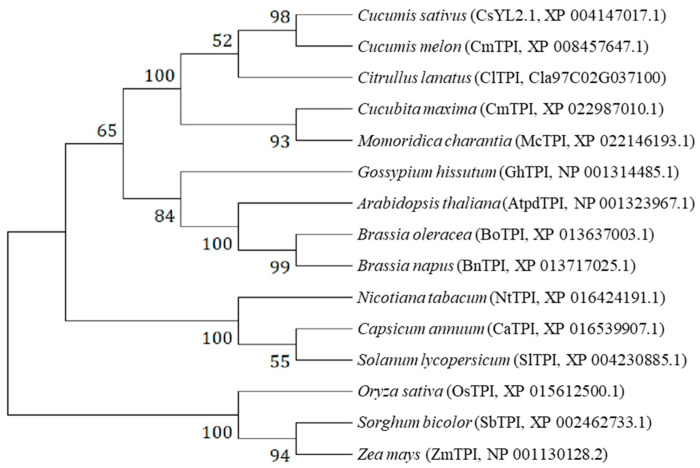
Phylogenetic tree of CsYL2.1 protein in cucumber and its orthologs in other species. There is no specific information on TPI or pdTPI for other species except that studies in *Arabidopsis thaliana* shows it to be pdTPI. The phylogenetic tree was constructed via the neighbor-joining method built in MEGA X. Numbers indicate the percentage of replicate trees in which the associated taxa clustered together in the bootstrap test (1000 replicates).

A phylogenetic tree was constructed on the basis of the amino acid sequences of CsYL2.1 and the orthologous proteins using the neighbor-joining (NJ) algorithm (Figure 3). The phylogenetic tree showed that CsYL2.1 and the orthologous protein in melon, which have the highest percentage of identity, clustered together. In addition, the orthologs from other cucurbitaceous plants (bitter gourd, pumpkin and watermelon) were located in the same evolutionary branch. At the same time, we found that the ortholog genes of all dicotyledons, including the solanaceous plants (tobacco, pepper, and tomato), and the cruciferous plants (rape, cabbage, and *A. thaliana*), are clustered together separately, while the ortholog genes of monocotyledons (rice, sorghum, and maize) are located in an outer branch.

### 2.6. Expression Pattern Analysis of CsYL2.1

**Figure 4:**
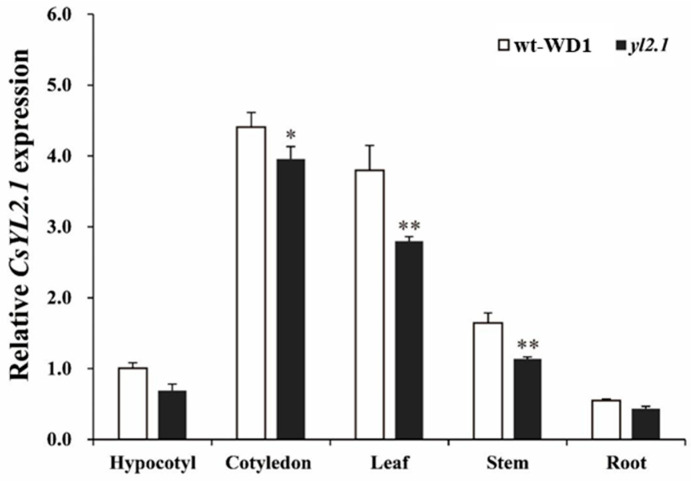
Relative expression of the *CsYL2.1* candidate gene. Relative expression of the *CsYL2.1* candidate gene in wt-WD1 and *yl2.1* was measured by qRT-PCR in the hypocotyls, cotyledons, leaves, roots, and stems. For each sample, three independent biological replications were performed. Data are means ± SD (*n* = 3). Transcript levels of *CsYL2.1* were normalized with *CsActin* and expression was shown relative to the hypocotyl of wt-WD1, the values of which were set as 1. Asterisks indicate statistically significant differences compared with wt-WD1 at ** *p* < 0.01 and * *p* < 0.05 by Student’s *t*-test.

The relative expression level of *CsYL2.1* in different tissues (hypocotyls, cotyledons, leaves, roots, and stems) in both of wt-WD1 and *yl2.1* was examined by qRT-PCR. The result showed that *CsYL2.1* was expressed widely in hypocotyls, cotyledons, leaves, roots, and stems (Figure 4). *CsYL2.1* was highly expressed in cotyledons and leaves in wt-WD1 and *yl2.1* plants. In hypocotyls, cotyledons, leaves, roots, and stems, *CsYL2.1* exhibited higher expression levels in wt-WD1 than in *yl2.1* (Figure 4).

### 2.7. CsYL2.1 Is Required for Chlorophyll Synthesis and Chloroplast Development

The pigment (total chlorophyll, Chl_a_ and Chl_b_) content of leaves in both wt-WD1 and *yl2.1* was measured. The result showed that the pigment content of *yl2.1* leaves was significantly lower than that of wt-WD1. The total Chl, Chl_a_, and Chl_b_ contents of *yl2.1* leaves were only 38.1%, 33.6%, and 37.1% of wt-WD1 leaves, respectively (Table 2). The result indicated that the yellow leaf phenotype of *yl2.1* was caused by the decrease in chlorophyll content in the leaves.

**Figure 5:**
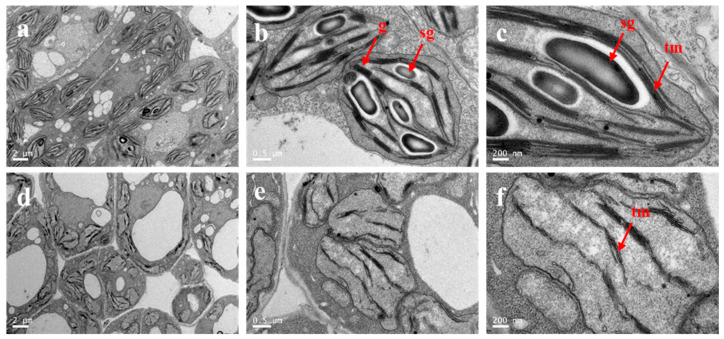
Chloroplast ultrastructure of *yl2.1* and wt-WD1. Chloroplast structure of wt-WD1 (**a**–**c**) and *yl2.1* (**d**–**f**). sg = starch granules; tm = thylakoid membranes; g = grana. Scale bar is shown in the figure.

To examine if *CsYL2.1* had an effect on chloroplast development, fresh leaves of wt-WD1 and *yl2.1* at the first true leaf stage were collected to perform transmission electron microscopy (TEM). Compared with wt-WD1, the *yl2.1* chloroplasts contained more abnormal stacked grana (g), a distorted thylakoid membrane system (tm), and swollen lumens (Figure 5). In addition, the *yl2.1* chloroplasts accumulate fewer starch granules (sg) in the chloroplast stroma than wt-WD1 (Figure 5a–f), suggesting that photosynthetic capacity in *yl2.1* is compromised.

### 2.8. CsYL2.1 Affects Photosynthetic Efficiency

Chlorophyll fluorescence parameters are a set of variables reflecting the “intrinsic” characteristics of plants and effectively representing the absorption, use, and dissipation of light energy by plants [10]. To confirm if *CsYL2.1* affected the photosynthetic capacity or not, we measured several chlorophyll fluorescence parameters, including minimum initial fluorescence (F_0_), maximum coefficient of fluorescence (F_m_), photochemical quenching (qP), the coefficient of non-photochemical quenching (NPQ), and the actual photosynthetic efficiency of photosystem II (YII). The results demonstrated that almost all the chlorophyll fluorescence parameters of *yl2.1* were lower than that of the wt-WD1 except for NPQ. The F_v_/F_m_ indicates the maximum photochemical quantum yield of photosystem II (PSII). Lower F_v_/F_m_ in *yl2.1* leaves demonstrated lower light filling efficiency of PS II. Besides, qP and YII in *yl2.1* leaves were significantly lower than that in wt-WD1. However, the NPQ in *yl2.1* leaves were significantly higher than that of wt-WD1 (Table 3). Lower qP in *yl2.1* reflected that the chlorophyll deficiency in *yl2.1* leads to the failure of the transformation of light quantum into chemical energy in photosynthesis. However, higher NPQ in *yl2.1* indicated that the *yl2.1* consumes excess light energy to protect the photosynthetic system in the way of heat dissipation. Fluorescence imaging analysis showed that the fluorescence of the *yl2.1* leaves was significantly shallower than that of the wt-WD1 leaves, indicating that the maximum light energy conversion efficiency of cucumber leaves was reduced in *yl2.1* (Supplemental Figure S7).

**Figure 6:**
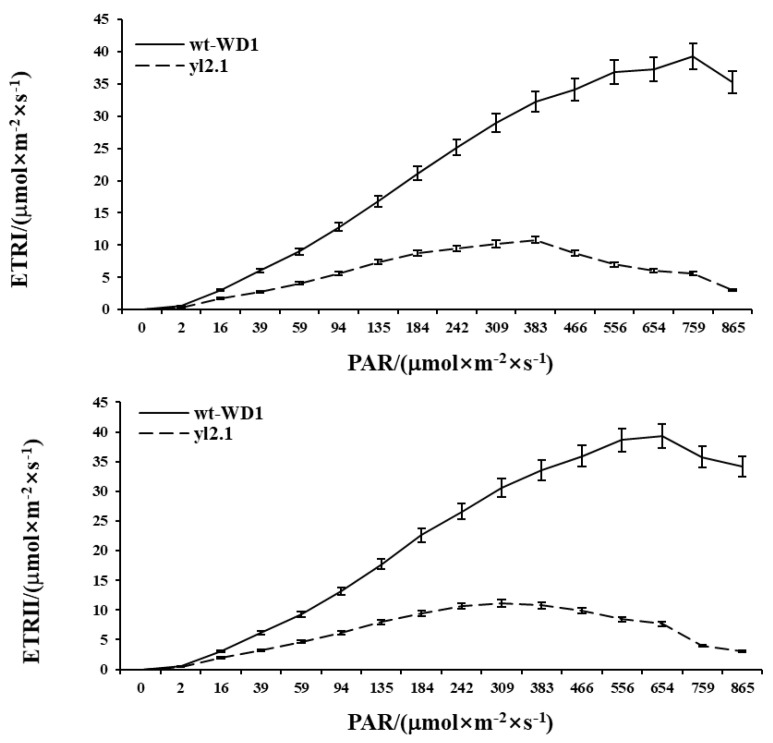
The PSI and PSII rapid light curve of *yl2.1* and wt-WD1. For each sample, five independent biological replications were performed. Each independent biological replication contains three different individuals. Three technical repeats were performed for each biological replication. The average of the three technical repeats was used as the data of one biological replication. Data are means ± SD (*n* = 5). ETRI indicated the rate of relative photosynthetic electron transport of photosystem I. ETRII indicated the rate of relative photosynthetic electron transport of photosystem II. PAR indicated photosynthetical active radiation.

The electron transport rate (ETR) can be used to analyze the light energy conversion rate. The ETR analysis showed that the slope of the rapid light curve and the maximum relative electron transfer rate (ETRmax) of *yl2.1* were significantly lower than that of wt-WD1, indicating that the electron transfer efficiency and the utilization rate of light energy of the mutant were significantly lower than that of the wt-WD1 (Figure 6).

## 3. Discussion

Leaf color mutation is a ubiquitous and easily identifiable phenotype in higher plants. Leaf color mutations usually affect the photosynthetic efficiency of plants, resulting in poor growth and economic losses. Leaf color is usually determined by photosynthetic pigment in the chloroplast [11]. Therefore, chlorophyll biosynthesis and chloroplast development are crucial to leaf color and photosynthesis. Thus, leaf color mutants are the ideal materials to explore the processes of the photosynthesis system, chlorophyll metabolism, and chloroplast development. The genetic modes of plant leaf color mutants are mainly divided into three types: nuclear heredity, cytoplasmic heredity and nuclear-plasmid gene interaction heredity, among which nuclear heredity is the most important genetic mode of leaf color. Mutations in any one of the nuclear genes or their regulators in chlorophyll biosynthesis and chloroplast development could result in variations of chlorophyll contents, leading to different leaf color mutations [12]. At present, there have been many studies on leaf color mutants including wheat (*Tricum aestivum* L.) [13], *A. thaliana* [14], rice [15], corn [16], carrot (*Daucus carota* L.) [17], cucumber [18], and many other crops. However, there are few studies on leaf color mutants in cucumber. In the present study, the *yl2.1* was identified in an EMS-mutagenized wt-WD1 cucumber population. The *yl2.1* seedlings exhibited yellow cotyledons and true leaves after germination and could not turn green during leaf development. The phenotype of *yl2.1* is different from the previously reported cucumber mutants. The new leaf mutant *yl2.1* is useful to study the chlorophyll biosynthesis and chloroplast development.

*Csa2G263900*, the only candidate gene for the *YL2.1* locus, encodes a predicted pdTPI. In *A. thaliana*, TPI catalyzes the reversible conversion of DHAP to GAP. Seedlings produce DHAP through glycerol catabolism, which is a by-product of lipid mobilization during seedling establishment [19]. A portion of the DHAP is likely to be transported into developing chloroplasts from the cytoplasm. Once into the chloroplast, DHAP is converted into GAP, catalyzed by pdTPI. DHAP and GAP are precursors for multiple metabolic pathways in the chloroplast and can affect chlorophyll synthesis and chloroplast development [19]. In *A. thaliana*, the *pdtpi* mutant exhibited chlorotic seedlings [19], indicating the pdTPI occupies a strategically important position in chloroplast metabolism and development. The deduced protein sequence of CsYL2.1 shares 77.8% identity with its homolog in *A. thaliana* (Supplementary Table S2). Dysfunction of CsYL2.1 is expected to lead to severely stunted and chlorotic seedlings, which is consistent with the phenotype observed in the *yl2.1*. Given the known functions of the protein, we concluded that *CsYL2.1* encodes a predicted pdTPI and plays an important role in chlorophyll synthesis and chloroplast development in cucumber.

The mechanism underlying leaf color mutation is complex. In cucumber, five genes related to leaf color mutation, namely *virescent leaf* (*v-1*), *virescent-yellow leaf* (*vyl*), *yellow-green leaf* (*ygl1*), *yellow plant* (*yp*), and *yellow young leaf* (*yyl-1*), have been cloned and characterized. These studies confirmed that the leaf color mutants were the result of the decrease in chlorophyll content caused by abnormal chloroplast development directly or indirectly. In *A. thaliana*, it has been shown that dysfunction of pdTPI prevented the conversion of DHAP to GAP in chloroplasts, resulting in DHAP accumulation and reduced GAP. DHAP is an important intermediate in lipid biosynthesis and glycolysis. It is a biochemical compound involved in many metabolic pathways, including the Calvin cycle in plants and glycolysis. GAP is the direct substrate for chlorophyll biosynthesis in the plastid [20]. A surplus of DHAP would limit ribulose 1,5-bisphosphate production, suppressing the Calvin cycle, chloroplast development, and photosynthetic capacity [21]. However, the decrease in GAP supply led to reduced chlorophyll biosynthesis and the chlorotic leaf phenotype. In this study, to determine whether CsYL2.1 affects chlorophyll biosynthesis and chloroplast development, we examined the chlorophyll content and chloroplast ultrastructure in the leaves of wt-WD1 and *yl2.1* at the first true leaf stage. Compared with wt-WD1, the contents of total chlorophyll, Chl_a_, and Chl_b_ in the *yl2.1* leaves decreased significantly (Table 2). Ultrastructural analysis by TEM showed that the mesophyll cells in *yl2.1* leaves contained fewer and abnormal chloroplasts (Figure 5). These results indicated that chlorophyll biosynthesis and development of chloroplasts was defective in *yl2.1.* Several chlorophyll fluorescence parameters indicated that the photosynthetic efficiency of *yl2.1* was significantly lower than that of wt-WD1. The decreased number of starch granules in the mesophyll chloroplasts of *yl2.1* leaves further confirmed that chlorophyll deficiency in *yl2.1* leaves leads to the inability to convert the light quantum into chemical energy. Taken together, *Csyl2.1* impaired chlorophyll biosynthesis and chloroplast development, resulting in etiolated and non-autotrophic seedlings. This study provided clues for us to further study the role of *CsYL2.1* in chlorophyll metabolism and chloroplast development and to explore the molecular mechanisms of this leaf color variation.

## 4. Materials and Methods

### 4.1. Plant Materials

An EMS-induced mutant *yl2.1* possessing yellow cotyledons and true leaves was obtained from the EMS-mutagenized wild-type inbred line WD1 (North China type, wt-WD1) cucumber population. Another wild-type 9930 (wt-9930), also known as “Chinese long”, is a North China fresh market-type inbred line with normal green cotyledons and true leaves and commonly used in modern cucumber breeding. Now, the wt-9930 has become an important research material because its genome was successfully sequenced in 2009. The mutant seedlings exhibited reduced growth and lethality at the fourth leaf stage. So, the homozygotes (+/+) and heterozygotes (*yl2.1/+*) were self-crossed and crossed with wt-9930. When the self-crossed progenies presented segregating phenotypes of green and yellow leaves, these plants and the F_1_ obtained by crossing these heterozygotes (*yl2.1/+*) and wt-9930 were retained to produce the F_2_ populations by self pollination. Only the F_2_ population with yellow leaves were selected for further analysis. All of the plant materials were then planted in the greenhouse of Shanghai Jiao Tong University.

### 4.2. Insertion–Deletion and Single Nucleotide Polymorphism Marker Development

All the markers, including insertion–deletion (InDel) and single nucleotide polymorphism (SNP) markers, were explored via whole-genome resequencing. The genomic DNA of *yl2.1* was extracted via the CTAB (Cetyl Trimethyl Ammonium Bromide) method [22]. The genome of the *yl2.1* was re-sequenced with the Illumina HiSeq™ 2000 platform (Biomarker Technologies). With 30-fold sequencing depth, all the clean reads were mapped to the ‘wt-9930′ genome sequence (version 2, http://cucurbitgenomics.org/organism/2, accessed 9 November 2020). The sequencing data were then compared with the reference genome sequence of wt-9930. The derived cleaved amplified polymorphic sequence (dCAPs) markers were developed with dCAPS Finder 2.0 (http://helix.wustl.edu/dcaps/dcaps.html, accessed 9 November 2020) [23]. All primers were designed by Primer Premier 5.0 software and synthesized by Sangon Biotechnology Company Limited (Shanghai, China). Information on all primers in this study is provided in Supplemental Table S3.

### 4.3. DNA Extraction and Molecular Marker Analysis

Genomic cucumber DNA was extracted from young leaves of wt-WD1, wt-9930, *yl2.1*, F_1_ samples, and each F_2_ individuals according to the cetyltrimethylammonium bromide (CTAB) method [23]. Polymerase chain reaction (PCR) was carried out with 10-μL samples containing ~80 ng of genomic DNA, 0.5 μM of each primer, 200 μM of dNTPs, 1× reaction buffer, and 0.5 U Taq DNA polymerase (Takara Biotechnologies, Beijing, China). PCR amplification was performed on a PCR machine (Applied Biosystems, Foster, CA, USA) via the following program: 94 °C for 5 min; 33 cycles of 94 °C for 15 s, 55 °C for 30 s, and 72 °C for 30 s; and 72 °C for 5 min. Products were separated on an 8% polyacrylamide gel by electrophoresis. After electrophoresis at 200 V for 1.5 h, the gel was separated from the plates and stained in 0.1% AgNO_3_ solution and transferred into a developing solution (1.5% sodium hydroxide, 0.4% formaldehyde) to reveal the silver-stained DNA bands.

### 4.4. Primary Mapping and Fine Mapping of YL2.1

The BSA method [24] was performed to analyze the linkage relationship between the markers and *YL2.1*. Wild-type and mutant DNA pools (W pool and M pool) were constructed by mixing equal amounts of DNA of five green-leaf plants and five yellow-leaf plants from the F_2_ population. Polymorphic InDel markers between wt-9930 and *yl2.1* were identified and applied to analyze the W and M pools. The *YL2.1* locus was primary-mapped by using 72 F_2_ individuals. Linkage analysis of the *YL2.1* locus with molecular markers was performed with Join Map 4.0 at a linkage of disequilibrium threshold of 4.0. In total, 1056 F_2_ individuals were used for fine-mapping of the *YL2.1* locus.

### 4.5. Gene Prediction and Candidate Gene Identification

Candidate gene prediction was based on the wt-9930 genome database (http://cucurbitgenomics.org/). The functions of the candidate genes were analyzed using the BLASTP tool from NCBI (https://blast.ncbi.nlm.nih.gov/Blast.cgi, accessed 9 November 2020). DNA fragments corresponding to candidate genes in this region were amplified from wt-WD1, wt-9930, and *yl2.1* genomic DNA with KOD PLUS DNA polymerase (Toyobo, Osaka, Japan) and sequenced. All primers used for sequencing are listed in Supplemental Table S3.

### 4.6. Phylogenetic Analysis and Alignment of CsYL2.1 and Its Homologous Proteins

A phylogenetic tree was constructed with the program MEGA X (www.megasoftware.net, 9 November 2020) based on the sequences of CsYL2.1 protein and its homologous proteins in different species downloaded from the NCBI database. The protein sequences used in phylogenetic tree construction were from following species: *Cucumis sativus* (CsYL2.1, accession No. XP_004147017.1), *Cucumis melo* (CmTPI, accession No. XP_008457647.1), *Momordica charantia* (bitter gourd, accession No. XP_022146193.1), *Cucurbita maxima* (CmTPI, accession No. XP_022987010.1), *Citrullus lanatus* (ClTPI, accession No. Cla97C02G037100), *Brassica napus* (BnTPI, accession No. XP_013717025.1), *Brassica oleracea* (BoTPI, accession No. XP_013637003.1), *Arabidopsis thaliana* (AtpdTPIaccession No. NP_001323967.1), *Gossypium hirsutum* (ChTPI, accession No. NP_001314485.1), *Solanum lycopersicum* (SlTPI, accession No. XP_004230885.1), *Capsicum annuum* (CaTPI, accession No. XP_016539907.1), *Nicotiana tabacum* (NtTPI, accession No. XP_016434191.1), *Oryza sativa* (OsTPI, accession No. XP_015612500.1), *Zea mays* (ZmTPI, accession No. NP_001130128.2), *Sorghum bicolor* (SbTPI, accession No. XP_002462733.1). Protein sequence alignment was performed with Clustal W and the neighbor-joining tree [25] was constructed, based on 1000 bootstrap replications in MEGA X.

### 4.7. RNA Extraction and Quantitative Real-Time PCR 

Cotyledons, hypocotyls, and roots of wt-WD1 and *yl2.1* were collected from seedlings at 10 days after germination (DAG). True leaves and stems were harvested at the first true leaf stage. Total RNA was extracted with an Omini Plant RNA Kit (DNase I) (CWBIO, Taizhou, China) according to the manufacturer’s instructions. First-strand cDNA was prepared with a HiFiScript cDNA Synthesis Kit (CWBIO, Taizhou, China). The quantitative real-time PCR (qRT-PCR) was conducted with Fast Start Universal SYBR Green Master Mix (ROX) (Roche, Shanghai, China) with a CFX96 TouchTM Real-time PCR system. The cucumber *CsActin* gene was used as the internal control [26]. Quantification of the relative changes in gene expression was performed via the 2^−ΔΔ*C*t^ method [27]. The qRT-PCR experiments were performed with three independent biological replications. Each independent biological replication contains at least three different individuals. Three technical repeats were performed for each biological replication. The qRT-PCR primers are listed in Supplemental Table S4.

### 4.8. Measurement of Chlorophyll Content

Fresh leaves (0.2 g) of *yl2.1* and wt-WD1 seedlings were cut into pieces and put into 50-mL tubes with 20 mL of extraction solution (acetone: 75% ethanol = 8:1 by volume) and kept in the dark for 2 days. The absorption of the extraction was measured in a WFZ UV-3802H spectrophotometer at 645, 663, and 470 nm wavelengths for Chla and Chlb. The concentrations of chlorophyll were calculated as described by Tang [28]. When the four true leaves unfurled, we harvested 10 yellow leaves and 10 normal green leaves at 9:00 to 11:00 a.m. on a sunny day to perform the experiment, which was repeated three times. Specific photosynthetic parameters, including the photosynthetic rate, intercellular carbon dioxide concentration, stomatal conductance and transpiration rate, were determined by a LI-6400 photosynthetic apparatus (LI COR, America). For determination of parameters for the red LED light source, the light intensity was set to 1000 μmol·m^−2^·s^−1^·photosynthetic photon flux density according to the LI-6400 manufacturers’ instructions. The experiments of chlorophyll content measurement were performed with five independent biological replications. Each independent biological replication contains three different individuals. Three technical repeats were performed for each biological replication.

### 4.9. Transmission Electron Microscopy

Fresh and fully expanded leaves from wt-WD1 and *yl2.1* seedlings were cut into small pieces and fixed with 1 mL of 0.1 M glutaraldehyde in a phosphate buffer at 4 °C for 12 h before they were placed in a vacuum at room temperature for 30 min, then rinsed and fixed with 1.0% osmium tetroxide at 4 °C for 1.5 h. The samples were dehydrated by a graded ethanol series and finally embedded in Epon 812 resin (Shell Chemical, America). Thin sections were sectioned with a Leica UC-7 ultra-microtome (Leica, Germany) and stained in uranyl acetate and lead citrate. Finally, the stained samples were viewed under a FEI Tecnai G2 Spirit Biotwin (Thermo Fisher Scientific, Shanghai, China) transmission electron microscope.

### 4.10. Measurement of Chlorophyll Fluorescence Kinetic Parameters

A WALZ company PAM2500 portable chlorophyll fluorescence spectrometer (Germany) was used to measure physiological parameters of the wt-WD1 and the *yl2.1* specimens, including the minimum initial fluorescence (F_0_), maximum coefficient of fluorescence (F_m_) and photochemical quenching (qP), the coefficient of photochemical quenching (NPQ), and photosystemIIactual photosynthetic efficiency (PSII). The wt-WD1 was selected as the control group; the *yl2.1* plants at the same growth stage were selected and functional leaves were treated in the dark with a dark-adaptation clamp (PAM2500 matching) for 50 min. Measurements were made according to the instructions of the PAM2500 portable chlorophyll fluorescence analyzer and the data were recorded. The experiments were performed with five independent biological replications. Each independent biological replication contains three different individuals. Three technical repeats were performed for each biological replication.

## Figures and Tables

**Table 1:** Segregation analysis of leaf color in F_2_ population.

Population	Total Number	Green Leaf	Yellow Leaf	Expected Ratio	χ^2^
F_1_	20	20	0	/	/
F_2_	1128	854	274	3:1	0.019

χ^2^ (0.05,1) = 3.84.

**Table 2:** Chlorophyll content of young leaves of the *yl2.1* and the wt-WD1 For each sample, five independent biological replications were performed. Each independent biological replication contains three different individuals. Three technical repeats were performed for each biological replication. Data are means ± SD (*n* = 5). Asterisks indicate statistically significant differences compared with the wild-type at ** *p* < 0.01 and * *p* < 0.05 by Student’s *t*-test.

Material	Chl_a_ (mg/g)	Chl_b_ (mg/g)	Total Chl (mg/g)	Chl_a/b_
wt-WD1	3.52 ± 0.02	1.31 ± 0.01	4.83 ± 0.08	2.69 ± 0.05
*yl2.1*	1.34 ± 0.03 **	0.44 ± 0.06 **	1.79 ± 0.03 **	3.03 ± 0.06 *

**Table 3:** Chlorophyll fluorescence kinetic parameters of young leaves of *yl2.1* and wt-WD1. For each sample, five independent biological replications were performed. Each independent biological replication contains three different individuals. Three technical repeats were performed for each biological replication. The average of the three technical repeats was used as the data of one biological replication. Data are means ± SD (*n* = 5). Asterisks indicate statistically significant differences compared with the wild-type at ** *p* < 0.01 and * *p* < 0.05 by Student’s *t*-test.

Material	F_0_	F_m_	F_v_/F_m_	qP	NPQ	Y(II)
wt-WD1	0.12 ± 0.03	0.48 ± 0.02	0.67 ± 0.01	0.67 ± 0.02	0.43 ± 0.10	0.45 ± 0.02
*yl2.1*	0.09 ± 0.01 **	0.29 ± 0.04 **	0.59 ± 0.05 *	0.47 ± 0.08 **	0.69 ± 0.17 **	0.18 ± 0.06 **

## Data Availability

The data presented in this study is available in supplementary material.

## Supplementary Materials

The following are available online at https://www.mdpi.com/1422-0067/22/1/322/s1, Figure S1: CDS sequence alignment of CsYL2.1 from the yl2.1 and the WD1 (WT), Figure S2: Amino acid sequence alignments of CsYL2.1 fromthe yl2.1 and the WD1 (WT), Figure S3: Phenotypic effects of non-synonymous single nucleotide polymorphisms (nsSNPs), Figure S4: Protein secondary structure analysis of CsYL2.1, Figure S5: Protein secondary structure analysis of Csyl2.1, Figure S6: Amino acid sequence alignments of CsYL2.1 in cucumber and its homologs in other species, Figure S7: The fluorescence imaging of the yl2.1 (top) and the WD1 (WT) (below). Table S1: Primer information for markers used in the present study for various purposes, Table S2: Primers used for qRT-PCR, Table S3: Information of 24 predicted genes in the 166.7kb region of the *yl2.1* locus, Table S4: Cucumber pdTPI homologous proteins used in phylogenetic analysis in this study.

## Abbreviations

F0	minimum initial fluorescence
F_m_	maximum coefficient of fluorescence
qP	photochemical quenching
SNP	single nucleotide polymorphism
PCR	Polymerase chain reaction
BSA	bulked segregation analysis
GAP	glyceraldehyde-3-phosphate
EMS	ethyl methane sulfonate
TEM	transmission electron microscopy
NPQ	non-photochemical quenching
ETR	electron transport rate
DHAP	dihydroxyacetone phosphate
Chla	chlorophyll a
Chlb	chlorophyll b
CTAB	cetyltrimethylammonium bromide
InDel	insertion–deletion
pdTPI	plastid isoform of triose phosphate isomerase

## References

[1] Nowaczyk M., Plumeré N. (2016). Short circuit at the chlorophyll. Nat. Chem. Biol..

[2] Bewley J.D. (1997). Seed Germination and Dormancy. Plant Cell.

[3] Chen M., Thelen J.J. (2010). The plastid isoform of triose phosphate isomerase is required for the postgerminative transition from heterotrophic to autotrophic growth in Arabidopsis. Plant Cell.

[4] Chen M., Thelen J.J. (2010). The essential role of plastidial triose phosphate isomerase in the integration of seed reserve mobilization and seedling establishment. Plant Signal. Behav..

[5] Miao H., Zhang S., Wang M., Wang Y., Weng Y., Gu X. (2016). Fine Mapping of Virescent Leaf Gene *v-1* in Cucumber (*Cucumis sativus* L.). Int. J. Mol. Sci..

[6] Gao M., Hu L., Li Y., Weng Y. (2016). The chlorophyll-deficient golden leaf mutation in cucumber is due to a single nucleotide substitution in *CsChlI* for magnesium chelatase I subunit. Theor. Appl. Genet..

[7] Song M., Wei Q., Wang J., Fu W., Qin X., Lu X., Cheng F., Yang K., Zhang L., Yu X. (2018). Fine Mapping of *CsVYL*, Conferring Virescent Leaf Through the Regulation of Chloroplast Development in Cucumber. Front. Plant Sci..

[8] Ding Y., Yang W., Su C., Ma H., Pan Y., Zhang X., Li J. (2019). Tandem 13-Lipoxygenase Genes in a Cluster Confers Yellow-Green Leaf in Cucumber. Int. J. Mol. Sci..

[9] Hu L., Zhang H., Xie C., Wang J., Zhang J., Wang H., Weng Y., Chen P., Li Y. (2020). A mutation in *CsHD* encoding a histidine and aspartic acid domain-containing protein leads to yellow young leaf-1 (*yyl-1*) in cucumber (*Cucumis sativus* L.). Plant Sci..

[10] Hazrati S., Tahmasebi-Sarvestani Z., Modarres-Sanavy S.A., Mokhtassi-Bidgoli A., Nicola S. (2016). Effects of water stress and light intensity on chlorophyll fluorescence parameters and pigments of *Aloe vera* L.. Plant Physiol. Biochem..

[11] Wang Q., Sullivan R.W., Kight A., Henry R.L., Huang J., Jones A.M., Korth K.L. (2004). Deletion of the chloroplast-localized Thylakoid formation1 gene product in Arabidopsis leads to deficient thylakoid formation and variegated leaves. Plant. Physiol..

[12] Tatsuru M. (2008). Recent overview of the Mg branch of the tetrapyrrole biosynthesis leading to chlorophylls. Photosynth. Res..

[13] Wu H., Shi N., An X., Liu C., Fu H., Cao L., Feng Y., Sun D., Zhang L. (2018). Candidate Genes for Yellow Leaf Color in Common Wheat (*Triticum aestivum* L.) and Major Related Metabolic Pathways according to Transcriptome Profiling. Int. J. Mol. Sci..

[14] Nagata N., Tanaka R., Satoh S., Tanaka A. (2005). Identification of a vinyl reductase gene for chlorophyll synthesis in Arabidopsis thaliana and implications for the evolution of Prochlorococcus species. Plant Cell.

[15] Li J., Wang Y., Chai J., Wang L., Wang C., Long W., Wang D., Wang Y., Zheng M., Peng C. (2013). Green-revertible Chlorina 1 (*grc1*) is required for the biosynthesis of chlorophyll and the early development of chloroplasts in rice. J. Plant Biol..

[16] Guan H., Xu X., He C., Liu C., Liu Q., Dong R., Liu T., Wang L. (2016). Fine Mapping and Candidate Gene Analysis of the Leaf-Color Gene ygl-1 in Maize. PLoS ONE.

[17] Nothnagel T., Straka P. (2003). Inheritance and mapping of a yellow leaf mutant of carrot (*Daucus carota*). Plant Breed..

[18] Cao W., Du Y., Wang C., Xu L., Wu T. (2018). Cscs encoding chorismate synthase is a candidate gene for leaf variegation mutation in cucumber. Breed Sci..

[19] Eastmond P.J. (2006). SUGAR-DEPENDENT1 encodes a patatin domain triacylglycerol lipase that initiates storage oil breakdown in germinating Arabidopsis seeds. Plant Cell.

[20] Lichtenthaler H.K. (1999). The 1-Deoxy-D-Xylulose-5-Phosphate Pathway of Isoprenoid Biosynthesis in Plants. Annu. Rev. Plant Physiol. Plant Mol. Biol..

[21] Timm S., Florian A., Fernie A.R., Bauwe H. (2016). The regulatory interplay between photorespiration and photosynthesis. J. Exp. Bot..

[22] Allen G.C., Flores-Vergara M.A., Krasynanski S., Kumar S., Thompson W.F. (2006). A modified protocol for rapid DNA isolation from plant tissues using cetyltrimethylammonium bromide. Nat. Protoc..

[23] Michael M.N., Edward T., Michael K. (2002). Web-based primer design for single nucleotide polymorphism analysis. Trends Genet..

[24] Michelmore R.W., Paran I., Kesseli R.V. (1991). Identification of markers linked to disease-resistance genes by bulked segregant analysis: A rapid method to detect markers in specific genomic regions by using segregating populations. Proc. Natl. Acad. Sci. USA.

[25] Saitou N., Nei M. (1987). The neighbor-joining method: A new method for reconstructing phylogenetic trees. Mol. Biol. Evol..

[26] Yang X., Zhang W., He H., Nie J., Bie B., Zhao J., Ren G., Li Y., Zhang D., Pan J. (2014). Tuberculate fruit gene Tu encodes a C2 H2 zinc finger protein that is required for the warty fruit phenotype in cucumber (*Cucumis sativus* L.). Plant J..

[27] Livak K.J., Schmittgen T.D. (2001). Analysis of relative gene expression data using real-time quantitative PCR and the 2(-Delta Delta C(T)) Method. Methods.

[28] Yanlin T. (2004). Change Law of Hyperspectral Data in Related with Chlorophyll and Carotenoid in Rice at Different Developmental Stages. Rice Sci..

