# Supplementary material for: A Mutation in CsYL2.1 Encoding a Plastid Isoform of Triose Phosphate Isomerase Leads to Yellow Leaf 2.1 (yl2.1) in Cucumber (Cucumis Sativus L.)
Source: Int J Mol Sci. 2020 Dec 30;22(1):322. doi: 10.3390/ijms22010322 (PMC7795558; doi:10.3390/ijms22010322)

Supplemental Figure S1. CDS sequence alignment of CsYL2.1 from the *yl2.1* and the WD1 (WT).

The full-length CDS of *CsYL2.1* was 921bp. The nucleotide mutation occurred at positions 649 from C to T in *yl2.1* (red box).

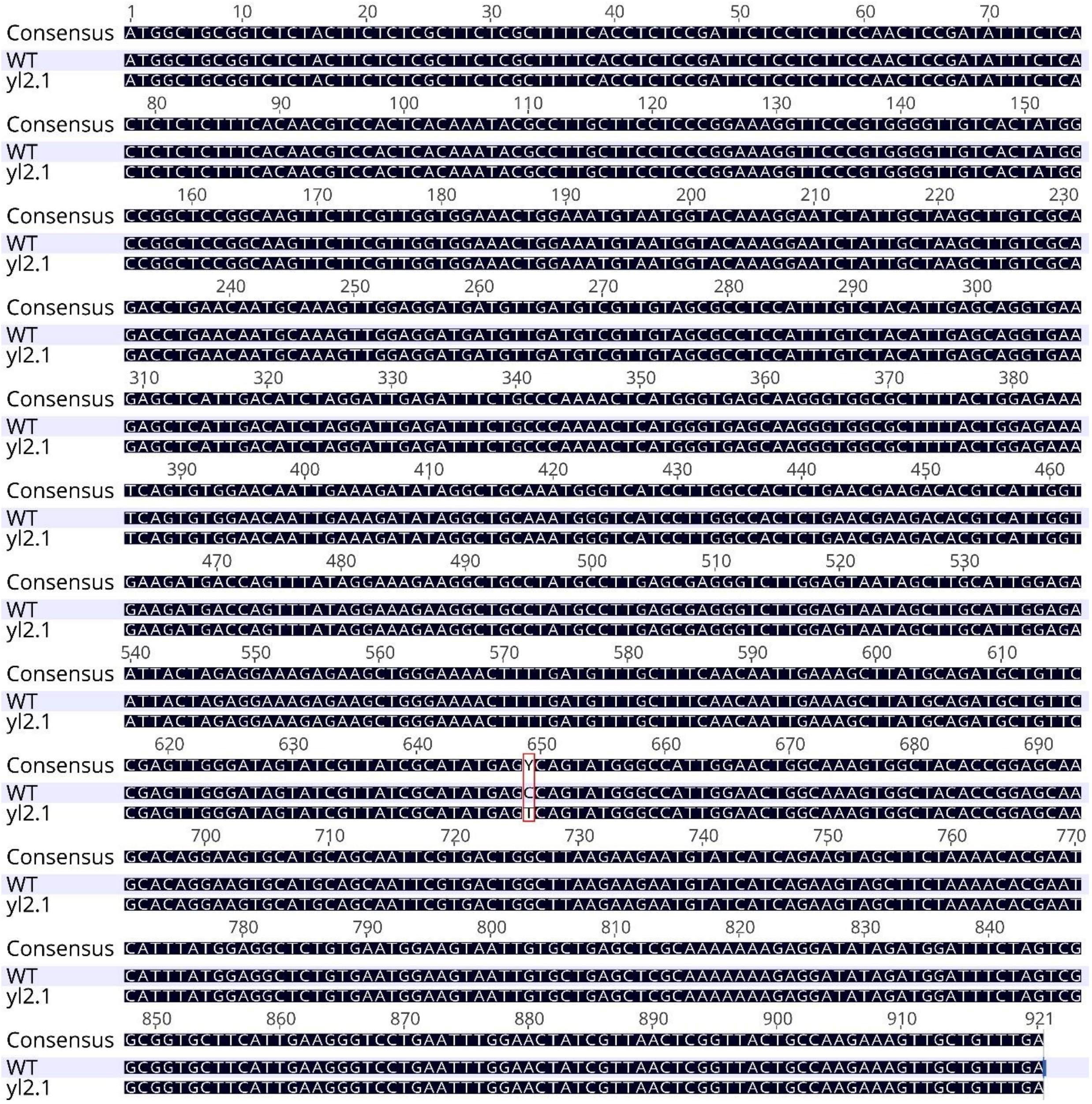

Supplemental Figure S2. Amino acid sequence alignments of CsYL2.1 fromthe *yl2.1* and the WD1 (WT).

The amino acid residue at position 217 was altered from Proline (P) to Serine (S) (red box).

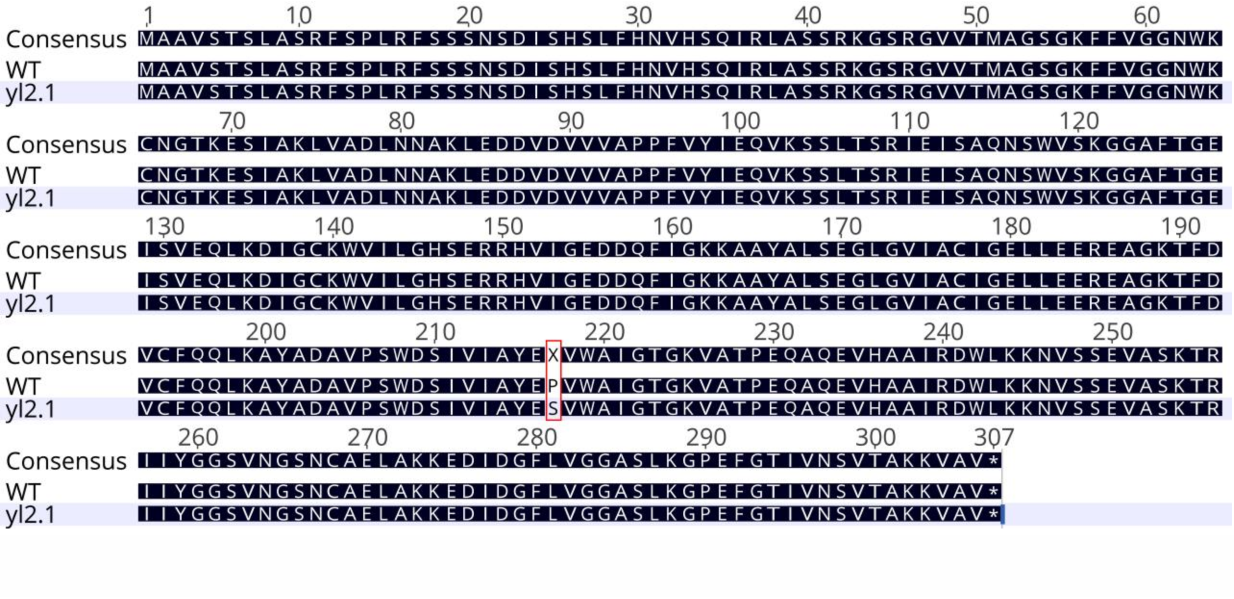

Supplemental Figure S3. Phenotypic effects of non-synonymous single nucleotide polymorphisms (nsSNPs)

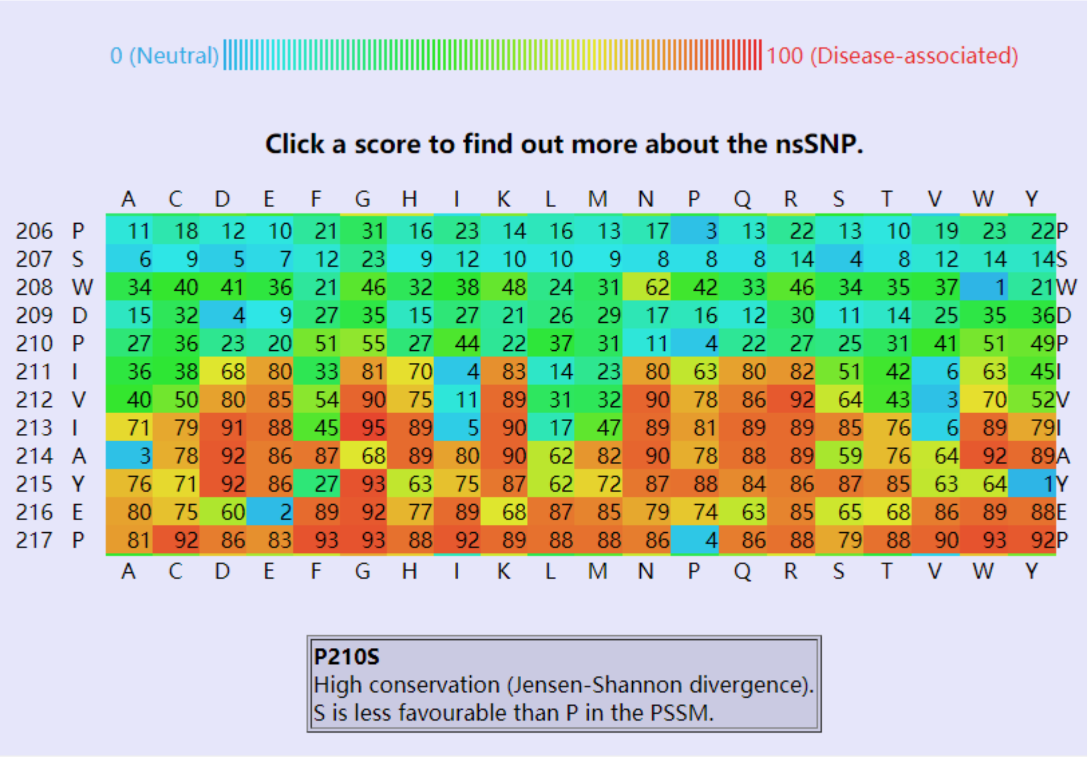

Supplemental Figure S4. Protein secondary structure analysis of CsYL2.1

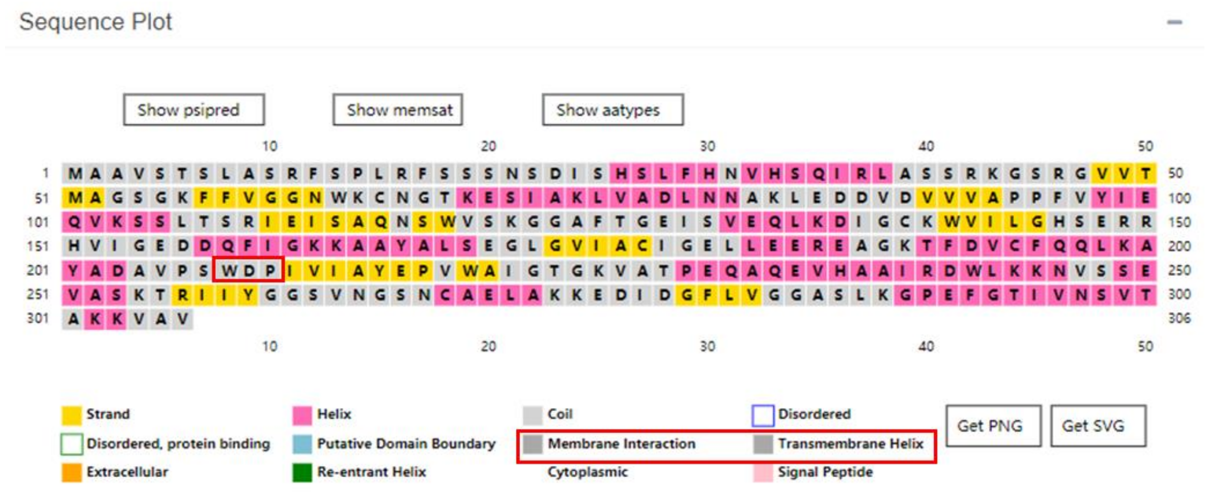

Supplemental Figure S5. Protein secondary structure analysis of Csyl2.1

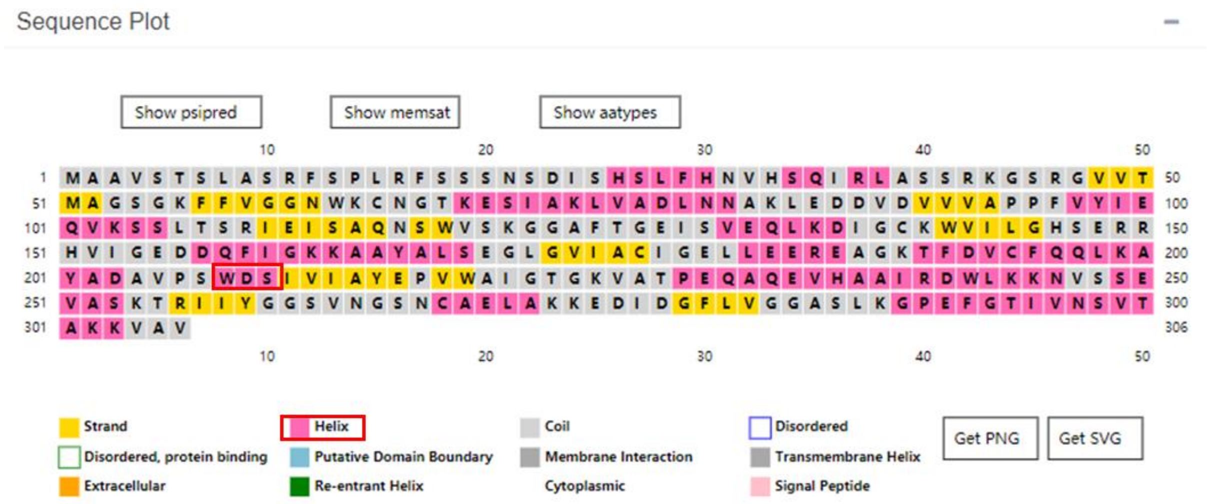

Supplemental Figure S6. Amino acid sequence alignments of CsYL2.1 in cucumber and its homologs in other species.

Red box indicates mutant site in the *yl2.1*.

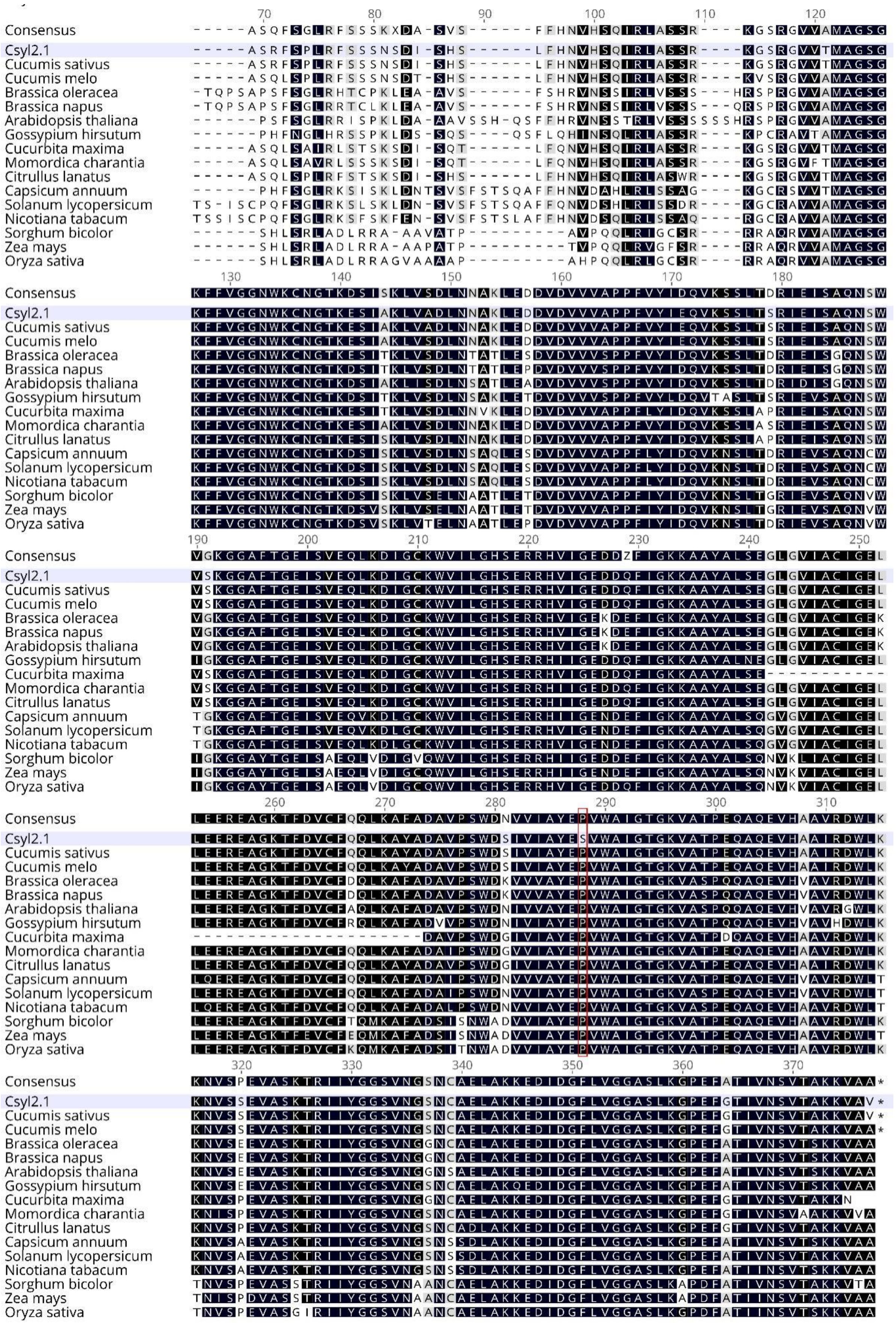

Supplemental Figure S7. The fluorescence imaging of the *yl2.1* (top) and the WD1 (WT) (below).

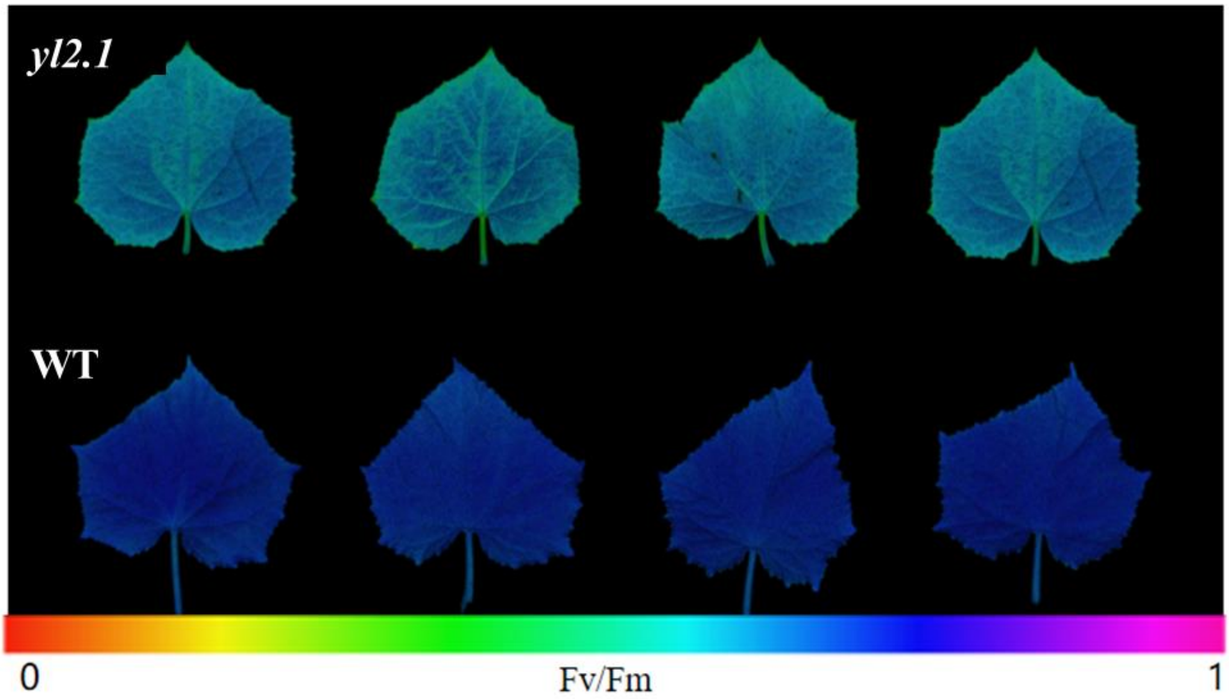

Supplement: Supplementary file 1 [file ijms-22-00322-s001.zip › Supplementary Figure(New).pdf]
